# Ecological Constraints on Tropical Forest Recovery Challenge the “Long-Term” Vision of the Kunming–Montreal Global Biodiversity Framework

**DOI:** 10.1093/biosci/biag007

**Published:** 2026-02-28

**Authors:** Erik Petter Axelsson, Ulrik Ilstedt

**Affiliations:** Department of Wildlife, Fish and Environmental Studies, Swedish University of Agricultural Sciences, Skogsmarksgränd, 907 36 Umeå, Sweden; Department of Forest ecology and Management, Swedish University of Agricultural Sciences, Skogsmarksgränd, 907 36 Umeå, Sweden

**Keywords:** biodiversity conservation, forest restoration, habitat heterogeneity, saproxylic organisms

## Abstract

The Kunming–Montreal Global Biodiversity Framework (GBF) outlines targets for protecting and restoring biodiversity by 2030, with the vision of “living in harmony with nature” by 2050. Although the 20-year vision clearly is aspirational, we emphasize that many forest attributes crucial for biodiversity—such as the abundance of large trees and the availability of dead wood—recover over much longer timescales. In a restored tropical forest, we estimate that it may require about 57 years to reach densities of large trees comparable to pristine forests (>75 cm DBH) and up to 144 years for the largest trees to attain similar sizes. Twenty years is also insufficient to restore dead-wood stocks, but as trees mature and die, they can replenish the pool of large dead wood essential for biodiversity. Achieving GBF goals will require policies and management strategies that recognize ecological timescales and commit to long-term restoration and protection.

## Main text

Global efforts to reverse biodiversity loss increasingly rely on large-scale ecosystem restoration as a central conservation strategy. Initiatives such as the Bonn Challenge, the Trillion Trees Campaign, and the UN Decade on Ecosystem Restoration reflect this growing commitment, now reinforced by the Kunming–Montreal Global Biodiversity Framework (GBF). The GBF outlines ambitious targets for protection and restoration of biodiversity by 2030 and a long-term Vision of “living in harmony with nature” by 2050. Although this Vision clearly is aspirational, it also raises important questions about what can realistically be achieved within such short policy timeframes, given the slow pace of ecological recovery. We emphasize that 20 years is a brief period from an ecological perspective—particularly in hyper-diverse tropical forests where structural complexity develops over centuries—and that meeting GBF goals will require aligning restoration ambitions with the biological timescales of forest recovery.

In many forest ecosystems, the structural features most critical for biodiversity develop over long time scales and cannot be recreated quickly. Among these, the largest—and typically oldest—trees play a particularly vital role in maintaining forest heterogeneity (Lutz et al. 2018, Mensah et al. 2020). Through their complex architecture and longevity, they provide habitats for a wide range of organisms, supporting much of the epiphyte biomass and diversity in tropical forests (Woods et al. 2015) and offering unique resources such as cavities used by birds, arthropods, amphibians, and reptiles (Pinho et al. 2020) and abundant food in the form of fruits, flowers, foliage, and nectar (Lindenmayer et al. 2012). Large trees further contribute to enhanced seed dispersal, moderate harsh microclimates, and create distinct microsites characterized by elevated soil nutrients and greater plant species richness. Consequently, they enhance biodiversity by providing a broader array of ecological niches (Lindenmayer et al. 2014, Mensah et al. 2020, Schweiger and Svenning 2020, Ali and Wang 2021, Caron et al. 2021). This resulting structural and environmental complexity underpins one of the most consistent patterns in community ecology—the positive relationship between habitat heterogeneity and species diversity (Hutchinson 1959, MacArthur and MacArthur 1961, Tews et al. 2004, Stein et al. 2014).

Despite their critical role for biodiversity, large-tree abundance is declining globally (Lindenmayer et al. 2012, Pinho et al. 2020) and in tropical forests specifically (Pinho et al. 2020, Xie et al. 2024). This decline has prompted calls for policy reform to better protect large trees and the biodiversity that they support (Lindenmayer et al. 2014). In parallel, restoring the structural complexity provided by large trees has become a central objective of forest restoration efforts, reflecting the same concern over the loss of key forest structures (Dickinson et al. 2016).

For many dependent species, the keystone role of large old trees continues for decades after death, as they become standing dead trees or large logs that constitute critical substrates for a wide range of species (Grove 2001, Ramírez-Hernández et al. 2019, Parajuli and Markwith 2023, Falconí-López et al. 2024). Dead wood in all its forms provides habitat for a substantial proportion of forest-associated organisms, with large and persistent pieces being particularly valuable (Thorn et al. 2020). Saproxylic organisms dependent on dead wood are among the most threatened groups in forest ecosystems (Grove 2001, Ramírez-Hernández et al. 2019, Parajuli and Markwith 2023, Falconí-López et al. 2024). Dead wood is especially important for rare species (Parajuli and Markwith 2023), and dead-wood availability thus serves as a strong indicator of a system’s conservation value (Grove 2001, Ramírez-Hernández et al. 2019, Parajuli and Markwith 2023, Falconí-López et al. 2024).

Global syntheses confirm the significance of dead wood for forest biodiversity across biomes but highlight tropical regions as seriously understudied (Seibold et al. 2015, Parajuli and Markwith 2023). Although tropical studies generally focus on the role of dead wood in carbon storage (Pfeifer et al. 2015, Meriem et al. 2016), a few have also demonstrated its importance for biodiversity (Falconí-López et al. 2024, Lackmann et al. 2025). As in other biomes, management history and degradation strongly influence dead-wood availability in tropical forests (Grove 2001, Pfeifer et al. 2015, Meriem et al. 2016). Together with the loss of large living trees, reductions in dead wood diminish the structural legacies that underpin forest resilience and long-term ecosystem functioning. The capacity of forests to resist structural change and retain these material legacies is crucial for sustaining ecosystem function after disturbance (Niedermaier et al. 2022).

Given the intrinsic limits on tree growth, lifespan, and the time required to attain large size, there are inherent constraints on what can be achieved within 20 years, even with exceptional restoration efforts. In addition to the degree of degradation (Zenner et al. 2013), the time elapsed since restoration is a key factor influencing successional trajectories and restoration success (Crouzeilles et al. 2016).

## Clues from natural succession

Natural succession following disturbance provides valuable insights into how long it takes forests to recover key ecological attributes such as biomass, tree species composition, structural complexity (Chazdon et al. 2025, Escobar et al. 2025), and dead-wood volume (DeWalt et al. 2003). Evidence from across the tropics shows that recovery is slow and varies among attributes. In dipterocarp forests of Borneo, the effects of selective logging on tree communities remained evident 35 years after harvesting (Hayward et al. 2021), and in northern Australia, biomass recovery required at least 50 years (Hu et al. 2020). Similar patterns emerge in Mexico, where secondary forests may require more than 100 years to regain their original species composition and biomass, and even longer to restore average tree size (Aryal et al. 2024). In Panama, incomplete recovery of tree species—particularly rare taxa—has been observed even after 120 years of succession (Elsy et al. 2024). For dead wood, regrowing tropical forests may need about 70 years to accumulate volumes comparable to those of old-growth stands (DeWalt et al. 2003). Recent large-scale chronosequence work in the Chocó rainforest further supports these long recovery trajectories, showing that maximum tree diameter, height, and aboveground biomass may require a century or more to approach old-growth reference levels, even under favorable landscape contexts with high surrounding forest cover (Escobar et al. 2025).

In South America and Africa, recovery rates differ markedly among forest attributes. Less-affected properties such as soil characteristics may rebound within about 20 years, whereas forest structure and tree diversity typically require 25–60 years to reach 90% of old-growth values, and biomass and species composition may take more than a century (Poorter et al. 2021). A global review of secondary tropical forests similarly found that tree biodiversity recovers after roughly 50 years, and biomass after about 80 years (Martin et al. 2013). These patterns confirm that recovery is a long-term process and that the most structurally and compositionally complex attributes are the slowest to return.

Although natural succession can rebuild many forest attributes, full recovery is not guaranteed. Fragmentation can restrict seed dispersal from intact forests, and altered environmental conditions may no longer support the original species pool. Because recovery times lengthen with increasing degradation (Poorter et al. 2021) and the degree of degradation strongly influences restoration trajectories (Zenner et al. 2013), vast areas of heavily degraded tropical land are unlikely to regain structural or biological integrity within 20 years. Active restoration interventions will therefore be essential if these ecosystems are to have any realistic chance of approaching predisturbance conditions. These findings highlight the limits of natural succession within policy-relevant timeframes and underscore the need for targeted management and restoration actions to accelerate structural and biological recovery.

## Clues from restoration and its potential to achieve targets

Assessing the extent to which forest restoration can help achieve biodiversity targets requires long-term field data, which are scarce because most restoration projects are relatively short-lived and monitoring seldom extends beyond 5–10 years (Banin et al. 2023). Consequently, few assessments cover the 20-year timeframe relevant to current policy goals (but see Hayward et al. 2021, Keller et al. 2023, Axelsson et al. 2024). Few studies have evaluated how restoration influences the structural components of large trees and the material legacies of dead wood in tropical forests over relevant temporal scales. Understanding how restoration contributes to long-term forest recovery therefore depends substantially on the limited number of studies that span multiple decades.

Three decades of postlogging monitoring in restored dipterocarp forests of Borneo indicate that the effects of selective logging on tree communities remain detectable at least 23–35 years after harvesting (Hayward et al. 2021). Although numerous studies show that enrichment planting can increase the diversity and abundance of late-successional tree species in the understory (Rivas-Alonso et al. 2021, Axelsson et al. 2024, Schubert et al. 2025), it remains unclear to what extent and over what timescales these interventions contribute to the development of structural complexity. For example, Rivas-Alonso et al. (2021) found that 10 years was insufficient for enrichment planting to increase the abundance of large trees compared with natural succession. Similarly, Schubert et al. (2025) observed that enrichment planting increased the number of late-successional tree species 16–18 years after treatment, but emphasized that “more time is needed” to determine whether these differences will translate into a mature, later-successional canopy.

The benefits of restoring dead-wood availability in depleted forests are well established in boreal and temperate systems (Hekkala et al. 2016, Sandström et al. 2019, Komonen et al. 2024), yet comparable evidence from tropical forests remains extremely limited. To our knowledge, only two such studies exist, both from Costa Rica. Fernandez Barrancos et al. (2022) found that restoration planting enhanced the accumulation of dead wood over 17 years of succession, from 1.7% to 41% of volumes observed in old-growth forests. Similarly, Lackmann et al. (2025) reported that restoration interventions can accelerate the recovery of fungal communities on dead wood in degraded tropical forests. These studies underscore the potential and the current limitations of tropical forest restoration to achieve targets within temporal scales relevant for GBF, highlighting the need for policy and management frameworks that support long-term monitoring and interventions aligned with ecological timescales.

## Case study in restored dipterocarp forest in Sabah, Borneo

We took advantage of a long-term restoration project—established when restoration ecology was still emerging as a research field—to assess how 20 years of postrestoration succession influenced the abundance of large trees as well as the amount and size distribution of downed dead wood. Before degradation by repeated selective logging of large trees (>60 cm DBH, representing the current cutting limit) and later by extensive forest fires during the 1983 El Niño, the site was characterized as mixed dipterocarp rainforest (Axelsson et al. 2024). The 1983 fire, which burned at an atypically large scale, fundamentally altered forest dynamics: Regeneration shifted from being governed by small-scale, gap-driven recruitment of late-successional tree species to rapid regeneration dominated by weedy vegetation and pioneer species across extensive areas (Woods 1989, Nykvist 1996). Late-successional tree species persisted only as scattered remnants (Axelsson et al. 2024). Restoration practices subsequently employed at the site combined assisted natural regeneration (ANR) with high-diversity enrichment planting.

**Figure 1.**
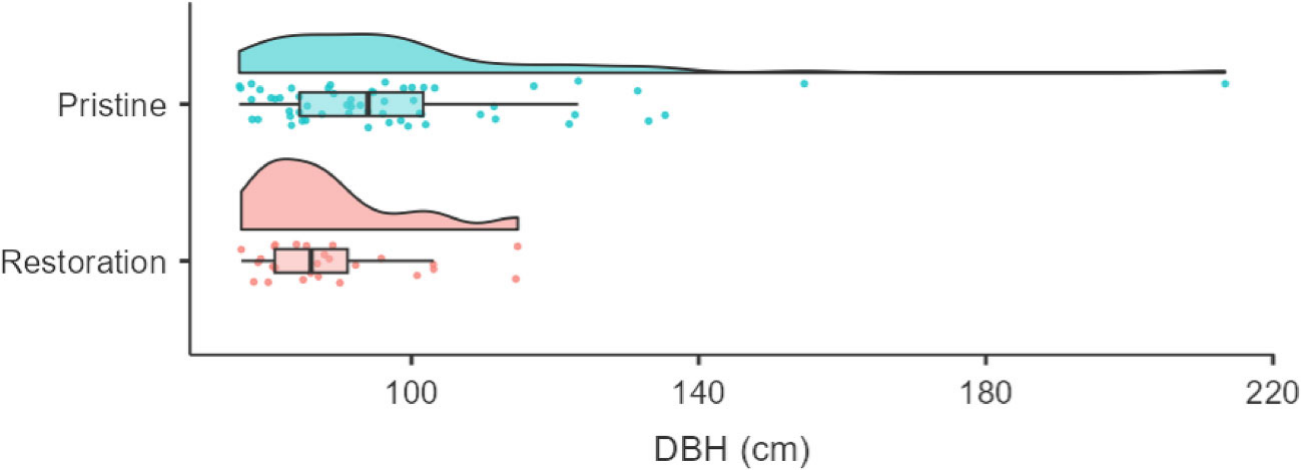
Size distribution of large trees (diameter at breast height > 75 cm) after 20 years of postrestoration succession compared to pristine reference forest. Data were generated from surveying 1619 trees across 4.25 ha of forests restored with enrichment planting and assisted natural regeneration within the INIKEA Sow-A-Seed restoration project (Axelsson et al. 2024) and 1398 trees across 4 ha in a nearby pristine forest in Sabah, Malaysia, Borneo.

**Figure 2.**
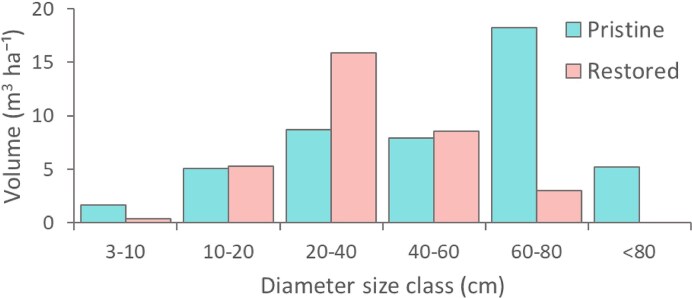
Distribution of dead-wood volume across diameter size classes in forests restored 20 years ago and in pristine reference forests. Data were generated from surveying dead-wood availability across 4.25 ha of forests restored with enrichment planting and assisted natural regeneration within the INIKEA Sow-A-Seed restoration project (Axelsson et al. 2024) and in a nearby pristine forest, in Sabah, Malaysia, Borneo.

In forests treated 20 years ago, approximately 1.7% (27 of 1619) of trees surveyed across 4.25 ha were large (DBH > 75 cm) compared with 4.1% (58 of 1398) in 4 ha of pristine forest. The size distribution of large trees in restored forests was also skewed toward smaller diameters, whereas pristine forests displayed a broader range, with the largest tree reaching 213 cm DBH (figure 1). Pristine forests also contained 9 trees (2.25 trees per ha) larger than the largest individual recorded in the restored forests. Moreover, downed dead wood in the restored forest had reached 71% of the volume found in pristine forest (33.1 versus 46.8 m³ ha⁻¹). Similarly, Fernandez Barrancos et al. (2022) reported that 17 years of postrestoration succession in Costa Rica yielded 41% of the dead-wood volume measured in old-growth forests. In our study, dead-wood size distributions in restored forests peaked in the 20–40 cm diameter class, whereas in pristine forests they were skewed toward larger diameter classes (60–80 cm; figure 2). This pattern aligns with findings from lowland tropical forests in Australia, where coarse woody debris more than 40 cm diameter was less abundant in logged and regrowing forests than in old-growth stands (Grove 2001).

## Predicting time to reach structural complexity of large trees and dead-wood legacies

Although 20 years of postrestoration succession clearly was insufficient to recreate the structural complexity associated with large trees or the abundance of large-diameter dead wood, we used these conditions to project future changes in structural and material legacies. Assuming that trees poised for ingrowth into the more than 75 cm DBH class survive and exhibit an average annual diameter increment of 0.5% (Miyamoto et al. 2021), it would take an additional 37 years to reach the same abundance of large trees as in the reference forest. By that time, mortality among large trees could also begin contributing to the accumulation of large-diameter dead wood. However, to achieve the full structural complexity of the pristine reference forest—based on the size of the largest trees—would require an additional 124 years. These estimates are consistent with successional studies of forests recovering from low-intensity land use (Poorter et al. 2021), despite the comparatively severe degradation of the site (Axelsson et al. 2024).

Although active restoration can accelerate recovery—for example, by planting late-successional species and thereby bypassing early seedling-establishment phases (Rivas-Alonso et al. 2021, Axelsson et al. 2024, Schubert et al. 2025)—our case study demonstrates that a 20-year timeframe is far from sufficient to reestablish the structural and material legacies characteristic of mature tropical forests. Although acknowledging that such estimates are context dependent and vary with the degree of degradation, our findings suggest that heavily degraded tropical forests undergoing restoration today may require approximately 57 years to attain densities of large trees comparable to that in pristine forests, and up to 144 years for the largest trees to reach similar sizes. Although these numbers derive from tropical systems, the same ecological principles likely apply broadly across forest biomes. Our results underscore the gap between ecological and policy timescales: Even with active interventions, structural attributes such as large trees and dead wood require many decades, often centuries, to recover. To meet the ambitions of the Kunming–Montreal GBF, restoration and protection policies must therefore be designed with these ecological realities in mind and be supported by long-term commitments that extend well beyond 2050.

## Policy and management recommendations for tropical forest conservation

The slow pace of recovery of large trees and dead wood demonstrated by our case study highlights that there are ecological limits to what the “restore” pillar of the Kunming–Montreal GBF can deliver within its stated timeframes. Recent work framing tropical forest recovery in terms of resistance and resilience similarly highlights that key structural attributes exhibit low resistance and slow resilience, implying recovery periods that far exceed current policy horizons (Escobar et al. 2025). To keep 2050 outcomes within reach, protecting existing large trees and old-growth structures must remain the first priority, whereas restoration should focus on accelerating structural recovery where feasible and where conserving remaining primary forests alone is insufficient to meet biodiversity targets. Restoration and reforestation efforts should prioritize natural forest regeneration using native tree species rather than short-rotation plantations, which generally comprise exotic species, lack structural complexity, and provide limited biodiversity benefits. High diversity enrichment planting should be employed to increase the value for restored forests for biodiversity, estimates suggests that at least 50 tree species are needed at a single site to provide the foundation for insect diversity in tropical forest (Axelsson et al. 2022). Using native species also strengthens ecological resilience and helps sustain the cultural and livelihood values that indigenous peoples and local communities (IPLCs) associate with forested landscapes, thereby promoting long-term stewardship and permanence (Axelsson and Grady 2022). Although our focus here is on restoring structural complexity and dead wood, ensuring that restored forests persist long enough for these gains to materialize is equally important. Short-term recovery can easily be reversed without sustained protection and incentives (Reid et al. 2019, Schwartz et al. 2020). Consequently, secure tenure and long-term financing mechanisms that match ecological recovery timescales are essential for permanence (Galatowitsch 2009, Le et al. 2012, Latawiec et al. 2015). Notably, experience from large, multidecadal restoration initiatives shows that sustained engagement in restoration in some cases can create incentives for conservation and lead to formal protection of recovering areas (Axelsson et al. 2024), turning restoration into a pathway for lasting biodiversity gains under the GBF. Enduring political commitments that extend beyond 2050 will be critical if global frameworks are to capture the full ecological timescales required for forest recovery and long-term biodiversity outcomes.

To translate these recommendations into practice, coordinated conservation, management, and policy actions must operate across spatial and temporal scales. In forest ecosystems, these scales range from the immediate protection of irreplaceable structures—such as existing large trees and old-growth forests—to multidecadal processes of structural recovery and the institutional frameworks needed to ensure durability and prevent reversal. Below, we outline a set of complementary strategies that together address these challenges, distinguishing actions that prevent further degradation from those that accelerate recovery and those that ensure long-term persistence.

### Conservation: Prevent further degradation and secure irreplaceable structures

At broad spatial scales, the most immediate and effective strategy for safeguarding forest biodiversity is the protection of remaining old-growth forests and large-tree refugia. Reinforcing legal protection and compliance for remnant high-integrity forests—through expanded protected areas (PAs), other effective area-based conservation measures (OECMs), and recognition of IPLC-governed areas—directly preserves living large trees and existing large-diameter necromass. These structures underpin forest heterogeneity and biodiversity and cannot be replaced within policy-relevant timeframes (cf. Lindenmayer et al. 2012, Lindenmayer et al. 2014).

In managed and production forests, conservation must also operate at the stand and landscape scales by retaining existing large trees and ensuring their recruitment into future cohorts. Introducing diameter-class retention rules—such as retaining all trees in the upper decile of the local size distribution—and mandating the retention of legacy trees after harvest can help maintain critical structural elements. Replacing single minimum-diameter cutting limits with stand-level harvest-volume caps and explicit retention thresholds better aligns forest use with long-term structural integrity (cf. Lindenmayer et al. 2014).

### Management: Accelerate recovery of structure and necromass

Where forests have already been degraded, management interventions can accelerate—but not fully substitute for—natural recovery processes. ANR and high-diversity enrichment planting with late-successional, long-lived species that are missing from regenerating cohorts can help speed diameter growth, canopy stratification, and structural differentiation. When combined with light-touch silvicultural treatments such as targeted weeding or liberation thinning, these approaches can shorten early successional bottlenecks, although their effectiveness remains context dependent and unfolds over decades (Rivas-Alonso et al. 2021, Axelsson et al. 2024, Schubert et al. 2025).

Recovery of dead wood, particularly in large diameter classes, represents an additional structural challenge. Where ecologically appropriate and safe, necromass availability can be increased through coarse woody debris creation—such as ring-barking selected mid-sized trees once minimum structural thresholds have been reached—or by strategically relocating large logs from impact zones to restoration areas to close habitat gaps (Tranberg et al. 2024). Any such interventions require strict protocols to minimize disease, pathogen, and provenance risks. These measures can accelerate habitat availability but cannot replicate the full diversity or longevity of dead wood generated by mature forests.

### Policy: Make gains durable, scalable, and resistant to reversal

To ensure that conservation and management gains persist over time, policy mechanisms must operate at national and international scales and extend beyond short political or economic cycles. Time-consistent incentives can reduce the risk of backsliding by linking concessions, subsidies, and financing to verified retention of large trees and demonstrable gains in large-diameter necromass. Incorporating no-rollback clauses and reversal penalties into contracts can help prevent short-term restoration gains from being undone by future market fluctuations.

Harvesting and infrastructure policies further influence long-term outcomes. In addition to mandating state-of-the-art reduced-impact logging, regulations should ensure sufficient recovery periods between harvests so that biomass and structural complexity can rebuild—specifically by requiring that harvest volumes remain below actual growth rates. Decommissioning or closing redundant roads can reduce fragmentation, poaching, and fire ignition risk, yielding near-term biodiversity benefits while supporting long-term recovery (Kleinschroth and Healey 2017).

Finally, monitoring systems must track the structural attributes that underpin biodiversity recovery. Embedding a small set of tractable indicators into national forest monitoring—such as the density of live trees above biome-specific diameter thresholds, the volume of coarse woody debris above defined size classes, and measures of vertical structural diversity—would complement existing GBF headline indicators and allow progress to be assessed against ecological, rather than purely areal, benchmarks.

Together, these strategies highlight the need to match conservation actions, management interventions, and policy frameworks to the spatial and temporal scales of forest recovery. Among these measures the strongest empirical support exists for protecting old-growth forests and large trees, including through retention forestry, whereas restoration interventions such as ANR, enrichment planting, and dead-wood creation offer promising but more context-dependent pathways for accelerating recovery (table 1). Aligning these approaches within durable policy frameworks is essential if global biodiversity targets are to translate into meaningful and lasting ecological outcomes.

**Table 1.** Summary of proposed policy, management, and conservation recommendations to support implementation of the Kunming–Montreal Global Biodiversity Framework (GBF) in tropical forests.

Domain	Primary aim	Mechanism/key action	Near-term effect (≤2030)	Trajectory toward 2050 goals	Core metric(s)	Evidence base
**Conservation**	Prevent further loss of large trees and necromass	Strengthen legal protection of remnant old-growth and large-tree refugia; expand PA/OECM coverage; secure IPLC-governed areas for long-term stewardship	Immediate reduction in risk of irreversible structural loss	Maintains old-growth structure and biodiversity integrity	Density of live trees ≥ X cm DBH; CWD ≥ Y cm	**Strong empirical and global synthesis evidence** (Lindenmayer et al. 2012, 2014)
**Management—retention forestry**	Retain and recruit large trees in production forests	Introduce upper-decile retention and legacy-tree mandates; cap harvest volume at stand level; maintain on-site necromass	Rapid gain in retained large-tree density	Accelerates transition to structurally complex managed forests; *reduces re-clearing pressure through on-site biodiversity value*	Percentage of large-tree retention; stand-level harvest volume	**Strong evidence** from long-term retention-forestry studies
**Management—structural restoration**	Accelerate recovery of canopy complexity and large-tree cohorts	Prioritize *native-species* enrichment and ANR in degraded forests; targeted weeding/liberation thinning *combined with tenure security, IPLC engagement, and cultural stewardship to promote permanence*	Faster diameter growth; improved vertical structure	Higher proportion of large trees and multilayered canopy by 2050; *reduced reversal risk where native species and local stewardship are maintained*	Mean diameter growth rate; canopy-height diversity; *forest persistence rate*	**Moderate and context-dependent evidence** (Rivas-Alonso et al. 2021, Axelsson and Grady 2022, Axelsson et al. 2024, Schubert et al. 2025)
**Management—necromass restoration**	Rebuild large-diameter dead-wood pools	Controlled creation of coarse woody debris (e.g., ring-barking) and strict-protocol relocation of large logs *within areas secured for long-term management*	Immediate habitat provision for saproxylic taxa	Self-replenishing necromass as cohorts mature; *permanent retention of structural legacies*	Volume of CWD ≥ Y cm; saproxylic species richness	**Emerging evidence; pilot-scale studies** (Tranberg et al. 2024)
**Policy—durability and incentives**	Secure long-term integrity and prevent reversal	Tie concessions and subsidies to verified retention; introduce anti-rollback clauses; lengthen rotations; decommission redundant roads; *support large-scale, multidecadal restoration that can generate incentives for conservation and formal protection*	Enforceable incentives and rapid compliance gains	Sustained biodiversity and carbon benefits through 2050; *enhanced permanence of restored forests*	Compliance rate; reversal incidents; rotation length; *area upgraded to higher conservation class*	**Growing policy evidence base; strong conceptual support**
**Policy—monitoring and financing**	Align monitoring, financing, and political commitments with ecological timescales	Embed large-tree and necromass metrics into national monitoring and GBF reporting; track *forest persistence and reversal rates*; establish *long-term financing mechanisms and commitments extending beyond 2050*	Transparent reporting and sustained funding	Robust feedback loop and financing horizon that match ecological recovery; *durable biodiversity outcomes beyond 2050*	Density ≥ X cm DBH; CWD ≥ Y cm; canopy-height distribution; *persistence index*	**High feasibility; growing examples of long-term conservation finance** (Galatowitsch 2009)

## Data Availability Statement

All quantitative data supporting the findings of this study are contained within the figures of the article and can be digitized directly from them. No additional datasets are required to reproduce the analyses.

## Author Biography

E. Petter Axelsson (petter.axelsson@slu.se) is affiliated with the Department of Wildlife, Fish and Environmental Studies, Swedish University of Agricultural Sciences, Skogsmarksgränd, Umeå, Sweden. Ulrik Ilstedt (ulrik.Ilstedt@slu.se) is affiliated with the Department of Forest Ecology and Management, Swedish University of Agricultural Sciences, Skogsmarksgränd, Umeå, Sweden.
